# The Novel Coronavirus – A Snapshot of Current Knowledge

**DOI:** 10.1111/1751-7915.13557

**Published:** 2020-03-06

**Authors:** Harald Brüssow

**Affiliations:** ^1^ Department of Biosystems Group of Gene Technology KU Leuven Leuven Belgium

## Abstract

Another animal to human transmission of a coronavirus occurred in December 2019 on a live animal market in the Chinese city of Wuhan causing an epidemic in China, reaching now different continents. This minireview summarizes the research literature on the virological, clinical and epidemiological aspects of this epidemic published until end of February 2020.

In late December 2019, Chinese physicians identified a series of pneumonia cases in Wuhan, an 11‐million inhabitant megacity and traffic hub in central China. The infections were epidemiologically associated with a seafood ‘wet’ market in the city. ‘Wet’ market means that live and dead animals are sold, raising the suspicion of another zoonotic virus infection that had spilled over into the human population similar to the severe acute respiratory syndrome (SARS) epidemic in 2002, which also started from a live animal market. In SARS, the viral source was traced back to a bat coronavirus with civets as potential intermediate host. The novel Wuhan virus figures under different names (2019‐nCoV for novel coronavirus in the research literature, SARS‐CoV‐2 by the International Committee on Taxonomy of Viruses, COVID‐19 as disease denominator by WHO), all names indicating that it represents a coronavirus. One might argue whether ‘new’ instead of ‘novel’ coronavirus is not a better term since it is not completely different, but related to the SARS coronavirus, while others find that SARS‐CoV‐2 suggests a too close relations with the SARS virus possibly leading to some confusion (Jiang *et al.*, 2020). As the Wuhan virus name changed, so changed the character of the epidemic and this overview can only provide a snapshot of the scientific research literature on this topic at the moment of this writing (29 February 2020).

## Virus isolation and genome sequencing

Within a record time of less than a month, the novel coronavirus was identified, isolated and sequenced by three groups of Chinese scientists. A large consortium coordinated by W. Tan from the Chinese Centers for Disease Control and Prevention (Chinese CDC) obtained eight complete viral genome sequences from nine patients either by direct RNA isolation and sequencing from bronchoalveolar lavage fluid (BALF) or from classical virus isolation on human airway epithelial cells (Lu *et al.*, 2020). The eight genomes showed sequence identity of 99.98%, i.e. only four nucleotides differed out of the 30 000 nucleotide‐long single‐stranded RNA genomes. This level of viral genomic identity isolated from different human subjects is unusual for an RNA virus that has been circulating for a long time in the human population. This observation suggests a recent single spill‐over event from an animal source into humans. Geneticists estimated that this event might have occurred in November 2019. The closest relatives of the new virus are coronaviruses isolated from bats in Eastern China, but they shared only 87.6% sequence identity with the new isolates. Therefore, viral taxonomists consider the novel coronavirus as a new viral species belonging to clade 2 of the Sarbecovirus group, acronym for SARS comprising betacoronaviruses. With the SARS virus, the new isolates shared even less, namely 79% sequence identity. While bats are still considered the most likely source for this novel coronavirus, bats were already hibernating at the time of onset of this epidemic and no bats were sold at the Huanan food market in Wuhan, suggesting an intermediate animal host where adaptation to human transmission might have occurred. Live hedgehogs, badgers, snakes and turtledoves were sold at the Huanan market. Claims were made, but were not (yet) substantiated that snakes or pangolins were intermediate hosts for creating the coronavirus by recombination events. W. Tan and colleagues, who now constitute the China Novel Coronavirus Investigating and Research Team, described subsequently the isolation of further coronaviruses from three patients in Wuhan who tested negative for 18 viral and four bacterial respiratory pathogens. These viruses were closely related to those of the earlier nine patients. In human airway epithelial cells, the cytopathic effect of this virus was a lack of cilium beating (Zhu *et al.*, 2020).

Another group led by Y.‐Z. Zhang from the zoonosis group of the Chinese CDC in Beijing determined the genome of a closely related coronavirus by deep meta‐transcriptomic sequencing of the bronchoalveolar fluid of a patient working at the seafood market of Wuhan (Wu *et al.*, 2020c). Analysis of the cell receptor interacting viral S (spike) gene showed indications of a recombination event. Protein modelling suggested the human angiotensin‐converting enzyme II (ACE2) protein as receptor.

Z.‐L. Shi from the Wuhan Institute of Virology, and collaborators, presented a detailed characterization of the viruses detected in seven patients from Wuhan suffering from severe pneumonia. Six of them were professionally associated with the local food market (Zhou *et al.*, 2020). Metagenomics of BALF yielded coronaviruses that shared 99.9% sequence identity between the patients and the other coronavirus isolates from Wuhan. Using primers corresponding to a region in the S gene, these scientists developed a sensitive and outbreak‐specific PCR test providing a diagnostic tool for the virus detection in BALF or alternatively oral swabs of patients. For one patient, the researchers demonstrated an IgM seroconversion followed by an IgG seroconversion when using ELISA technique with the viral nucleocapsid as target antigen. Three patients showed specific IgM responses indicating an acute infection. This serological evidence is important since the full set of Koch’s postulates for the identification of a new pathogen has not yet been fulfilled. From one patient, they isolated a virus that caused cytopathic effects on the established Vero cell line after 3 days of incubation. The virus‐infected cells were stained with specific antibodies and were observed by fluorescence microscopy, allowing the establishment of a virus neutralization test. All patients developed neutralizing serum antibodies. While HeLa cells could not be infected with the novel coronavirus, this cell line became susceptible when expressing the ACE2 protein of human, bat and civet (but not of mouse) origin indicating that this novel coronavirus uses this protein as a cell receptor, as did the SARS coronavirus from the 2002 epidemic.

Zhang *et al. *(2020a) compared 27 novel coronavirus genomes isolated from patients in three Chinese cities (Wuhan, Zhejiang and Guangdong) and Thailand, all of whom had contact to Wuhan. The genomes were very similar but could still be classified into six genogroups, suggesting that mutations had accumulated within patients during the current outbreak. Curiously, the most basal genogroups were detected in Guangdong, and not Wuhan, isolates. Another group did an in‐depth annotation of the genomes of the novel coronaviruses with SARS virus and identified 380 amino acid substitutions affecting all predicted proteins of the viral genome (Wu, Peng *et al.*, 2020b).

## Clinical observations

One study focused on the cases of 41 patients hospitalized in Wuhan before January 2, all of whom had laboratory‐confirmed 2019‐nCoV infection (Huang*et al.*, 2020). At this earliest phase of the outbreak, patients were mostly males (73%), half of whom had underlying diseases and 66% of whom were exposed to the Huanan seafood market. Common presenting symptoms were fever (98%), cough (76%) and myalgia or fatigue (44%). Dyspnoea (laboured or difficult breathing) developed in 55%; acute respiratory distress syndrome (ARDS) was seen in 29%; 32% of patients needed to be transferred to an intensive care unit (ICU); and 15% died. The patients showed lymphopenia (a reduction of lymphocytes in the circulating blood) and signs of a ‘cytokine storm’. A follow‐up study investigated 99 patients at Jinyintan Hospital in Wuhan between January 1 and January 20. All 99 patients had PCR‐confirmed 2019‐nCoV infection (Chen *et al.*, 2020). During this next phase of the epidemic, fewer patients had had an exposure to the Huanan seafood market (49%), but they were still predominantly male (67%). The average age was 55 years, and again, half of them suffered from chronic diseases. The predominant clinical manifestations in these patients were fever (83%), cough (82%) and shortness of breath (31%). Imaging techniques showed bilateral pneumonia in 75% of the cases. Seventeen per cent developed ARDS, which worsened in 11%, leading to death from multiple organ failure. In a third report, 138 patients with confirmed novel coronavirus infection were admitted between January 1 and 28 at Zhongnan Hospital of Wuhan (Wang *et al.*, 2020c). This report differed from the previous two in important respects. In this cohort, only 9% of the patients reported having had an exposure to the Huanan seafood market, and the gender ratio was not significantly biased. The presenting symptoms were fever (99%), fatigue (70%) and dry cough (59%), followed by anorexia, myalgia and dyspnoea. Bilateral shadows, or ground glass opacities, were revealed by imaging techniques in the lungs of all patients. Overall, 26% of the patients needed a transfer to ICU and 4% died. Half of the patients showed comorbidities (hypertension, cardiovascular disease and diabetes). Most notably, 41% were possibly infected in the hospital, including 40 healthcare workers. Until February 9, only nine cases of 2019‐nCoV infections were reported in infants under 1 year in China, all of whom had had infected family members. All infants had a mild form of disease (Wei *et al.*, 2020).

## Epidemiology

For planning public health measures, basic transmission data for the novel coronavirus are essential. The first 425 confirmed cases from Wuhan provided data for a first epidemiological analysis (Li *et al.*, 2020a), but it should be kept in mind that the virus might evolve during the epidemic and change its properties. Over the three early phases of the infection (< Jan1, Jan1‐11, Jan 12‐22), no change in average age was seen (56–61 years); no cases < 15 years of age were observed. Male dominance among the patients disappeared, and the degree of ‘wet’ market exposure in the patients dropped over time. In addition, patient contact with persons showing respiratory symptoms was reported in fewer than 30% of the cases. These scientists calculated a mean incubation period of 5.2 days displaying a long tail (95th percentile: 12.5 days). Comparisons of index cases and secondary cases in five clusters yielded a ‘serial interval’ of 7 days. From onset of illness to a medical visit, and then to hospital admission, 5 and 12 days elapsed respectively. The Wuhan epidemic showed a doubling time of 7 days. From the cluster analysis, a basic reproduction number of 2.2 was estimated, i.e. each case led on average to 2.2 new infections. Another report investigated a case of a family from Shenzhen visiting relatives in Wuhan (Chan *et al.*, 2020). One Wuhan relative had developed fever, cough and dyspnoea four days before the arrival of family members from Shenzhen. Four further relatives developed respiratory symptoms which led to hospitalization in two. From the six Shenzhen family visitors, the four adults developed symptoms (fever, cough, weakness and diarrhoea) during the 5 days of their Wuhan visit, while two children remained unaffected. Notably, a family member who remained in Shenzhen contracted the disease four days after the return of the visitors from Wuhan to Shenzhen. The data are evidence for efficient human‐to‐human virus transmission. The virus was detected in most nasopharyngeal and half of the throat swabs, a single serum sample, but no urine or stool sample of this family cluster. Of importance for easy sampling and virus diagnostics, 90% of hospitalized patients from Hongkong were positive for PCR virus detection in self‐collected saliva (Te *et al.*, 2020). The viral load ranged from 10^3^ to 10^8^ viral copies per ml saliva and decreased over the duration of hospitalization, but in one patient, it was still detectable 11 days after hospitalization. Another study extended the epidemiological analysis to 88 infected patients living outside of Wuhan, but who had a recent travel history to Wuhan (Backer *et al.*, 2020). This cohort allowed an estimation of the mean incubation period to 6.4 days. Upper estimates range up to 11 days, which is important to determine the appropriate duration of quarantine.

## Spread

The novel coronavirus is spreading rapidly. On January 19, the first patient was reported on the West Coast of the United States. The patient developed respiratory symptoms after a family visit to Wuhan (Holshue *et al.*, 2020). During hospitalization, the patient developed diarrhoea and the stool was weakly virus‐positive. This observation is important since it could indicate a gut tropism in addition to lung tropism, not unusual for coronavirus. One might therefore count on a changing symptomatology in the unfolding of the current epidemic, necessitating public health measures against faecal–oral transmission (Danchin *et al.*, 2020). This scenario is not unlikely, since the ACE2 host receptor for the novel coronavirus is highly expressed on gut enterocytes (Liang *et al.*, 2020). Some change in symptomatology was already seen for Chinese patients outside of Wuhan. Among the first 2019‐nCoV patients from Beijing, upper airway congestion was the dominant finding (Chang *et al.*, 2020a). Disease in 62 laboratory‐confirmed patients from Zhejiang province showed mostly mild to moderate disease; 9% had diarrhoea, and only one patient needed ICU (Xu *et al.*, 2020). When analysing 72 314 cases, an overall case fatality rate (CFR) of 2.3% was calculated. However, when the analysis was limited to cases outside of the Hubei province (where Wuhan is located), the CFR was only 0.4% (Wu and McGoogan, 2020), possibly suggesting change of the epidemic to milder diseases with larger chains of transmission (Vetter *et al.*, 2020).

On January 24, two citizens of Germany developed symptoms and became 2019‐nCoV‐positive after meeting a Chinese business partner, who only became ill on the flight back to China. Two further subjects developed symptoms who had contact with one of the infected Germans notably before this person developed symptoms. This report suggests that infected persons can infect contact persons during the incubation period (Rothe *et al*, 2020).

Wu and Leung (2020a) estimated the national and global spread of the novel coronavirus on the basis of the air and train traffic data from and to Wuhan, accounting for the strict metropolitan‐wide quarantine measure imposed on Wuhan on January 24 by using an infection metapopulation model. According to this calculation, sufficient numbers of infected subjects had already been exported to major Chinese cities (900 infected subjects to five cities) to start secondary local epidemics. The peak of the Wuhan epidemic was predicted for April 2020 and that of local epidemics peaks in other Chinese cities would lag by 1–2 weeks. If transmissibility could be reduced by 25% through restrictions of people’s mobility, then the magnitude of the epidemic could be reduced by 50% and the peak of the epidemic would be delayed by one month. One major unknown factor of this model is the seasonality of the novel coronavirus infections, respiratory infections being typical winter infections declining with the warming of the seasons. Research, available only as preprint (Lai *et al.*, 2020), estimated that more than 800 infected subjects from Wuhan travelled to international destinations, with Thailand and Japan leading the list. Public health measures must therefore also be taken in these countries to contain the international spread of the epidemic. The epidemic has also reached Europe with an unexplained focus in Northern Italy. Particularly, worrisome will be the spread of infections to African countries where many regions have close economic connections with China, but insufficient hospital and public health resources to assure efficient containment measures (Gilbert *et al.*, 2020). Risk of transmission to Africa and South America is, however, by some epidemiologists considered to be low (Haider *et al.*, 2020). It is currently unclear whether transmission will be muted in agro‐economies with lower population densities.

## Treatment

So far, Chinese physicians have developed an empirical treatment and triage algorithm based on their experience with patients from Wuhan (Zhang, *et al.*, 2020b). This triage scheme proposes a separation of patients into those receiving home treatment and those receiving treatments for regular community acquired pneumonia. Suspected viral pneumonia cases are tested for the novel coronavirus under isolated observation; in case of dyspnoea and hypoxia development, supplemental oxygen supply will be given. The viral pneumonia patients were treated with arbidol, approved in Russia and China for influenza treatment. When the diagnosis of the novel coronavirus has been confirmed, a patient has been transferred to a specially designated hospital. The efficacy of arbidol against coronavirus is not, however, well substantiated scientifically or clinically.

Emergency conditions call for a need for speed in drug development. A promising approach is the repurposing of drugs tried against coronaviruses from previous outbreaks with SARS or Middle East Respiratory Syndrome (MERS) coronavirus (Li and De Clercq, 2020). MERS, in contrast with SARS, is a coronavirus zoonosis of likely bat origin with camels as intermediate hosts that is still circulating. The four non‐structural proteins that were preclinically explored as antiviral targets against SARS and MERS coronaviruses are reasonably well conserved in the novel coronavirus, raising hopes for this approach. However, patient enrolment for a MERS treatment trial with lopinavir (an antiretroviral proteinase inhibitor)/ritonavir (cytochrome P450 inhibitor to prolong the half‐life of lopinavir) and interferon‐β1b (MIRACLE) is still ongoing. Numerous clinical trials have been registered in China to test different compounds or combinations of compounds against the new coronavirus infection. Test drugs range from antiviral nucleotide analogs over viral protease inhibitors to traditional Chinese herbal medicine (e.g. *Forsythia* derivative Lian qiao) (Maxmen, 2020). It is important to start the tests now to get the informative patients enrolled and tested before the current epidemic stops. WHO suggests a shared standard clinical protocol for these trials to make the outcomes comparable.

With virus neutralization tests now at hand, Chinese researchers have already done *in vitro* efficacity tests against the novel coronavirus (Wang *et al.*, 2020a). The most promising effects were observed with the nucleotide analog remdesivir and the anti‐malaria compound chloroquine. Both showed inhibition of the novel coronavirus in the low micromolar concentration range. Remdesivir acts on viral RNA transcription at the postviral entry level, while chloroquine needs to be applied at the beginning of the infection; chloroquine’s action on lysosome membranes might interfere with the viral entry–fusion events. It is of note that the nucleotide analog remdesivir had a better activity against MERS coronavirus than the protease inhibitor lopinavir combined with ritonavir, and this both in cell culture and in mouse infections. In the mouse MERS infection model remdesivir improved pulmonary function, reduced lung viral loads and decreased severe lung pathology (Sheahan *et al.*, 2020). In addition, remdesivir given prophylactically to MERS virus‐challenged rhesus monkeys significantly reduced viral titres in the lung and prevented lung pathology as assessed by histology or by X‐ray radiographs when compared to control animals (de Wit *et al.*, 2020). Treatment with remdesivir 12 h after viral challenge had a more limited protective effect in rhesus monkeys. In early February, two randomized, placebo‐controlled clinical trials testing the therapeutic efficacy of remdesivir were started in China. One trial will enrol 308 patients with mild or moderate novel coronavirus disease (ClinicalTrials.gov: NCT04252664) and the other 453 patients with severe disease (ClinicalTrials.gov: NCT04257656). The enrolment is planned to be completed by end of April and May 2020 respectively. The importance of evidence‐based treatments proven in controlled clinical trials must be stressed since in the SARS epidemic untested drug treatment seems to have done more harm than no treatment (A. Danchin, personal communication). Therapeutics directed against immunopathological host responses might have a treatment value in view of the ‘cytokine storm’ seen in some patients with novel coronavirus infections.

## Prevention and control

Vaccines against MERS and SARS are currently not available. As in the case of the SARS epidemic, the current epidemic must be fought with public health measures. As a first measure, the Chinese authorities have closed the ‘wet’ seafood market in Wuhan. Due to the close contact of live animals with humans, these markets offer ideal conditions for the transmission of zoonotic infections. After two coronavirus infections having emerged from Chinese ‘wet’ food markets, strict hygiene measures are clearly warranted. However, consuming meat and other products of wild animals has a long tradition in China. There is a philosophy of medicine food homology, (‘eaten when hungry is food, eaten when ill is medicine’) such that in China pangolin products, for example are reputed to help against rheumatism; ‘meridian obstruction’; liver disease; and to improve eyesight (Li and Li, 2020b). More recently, consumption of meat of wild animals has become a sign of social status. It will not be easy to eradicate these false medical beliefs. Interdiction might create black markets; therefore, some scientists recommend strictly controlling, instead of forbidding, this market. Protecting the healthcare workers against nosocomial infection is another urgent need, since patient care will already represent a heavy burden to the health system and any reduction in health personnel would cause further problems. Face masks are insufficient protection for them; N95 masks, goggles and protective gowns are needed for medical personnel (Chang *et al.*, 2020b). For the general public, frequent hand washing, cough and sneezing etiquette and wearing masks when visiting public places are recommended (Wang *et al.*, 2020b). However, scientific evidence is lacking for the effectiveness of wearing masks by the uninfected person in contrast to the proven effectiveness of hand washing against respiratory infections. The SARS epidemic was contained by means of syndromic surveillance; isolation of patients; and quarantine of contacts. These measures will also play an important role in the current epidemic which, due to its sheer size, will necessitate more draconian measures such as limiting the movement of persons to and from hotspots of infection, resulting in the lockdown of Wuhan and of particular small cities in Italy and Germany. WHO has declared the novel coronavirus epidemic as a public health emergency. This expanding epidemic will be a stress test for existing health systems, including those of industrialized countries. It should also be a further motivation to strengthen fundamental research in trans‐species viral infections and on potential zoonosis impacts, particularly from bats, under changing environmental conditions. From the viewpoint of citizens, when it comes to the protection of lives, one might ask whether one should not put the same spending on public health and preventive research as on the spending on defence budgets.

## Conflict of interest

None declared.
